# The different outcomes between breast-conserving surgery and mastectomy in triple-negative breast cancer: a population-based study from the SEER 18 database

**DOI:** 10.18632/oncotarget.13976

**Published:** 2016-12-16

**Authors:** Qing-Xia Chen, Xiao-Xiao Wang, Pei-Yang Lin, Jie Zhang, Jun-Jing Li, Chuan-Gui Song, Zhi-Ming Shao

**Affiliations:** ^1^ Department of Breast Surgery, Affiliated Union Hospital, Fujian Medical University, Fuzhou, China; ^2^ Department of Breast Surgery, Key Laboratory of Breast Cancer, Fudan University Shanghai Cancer Center, Shanghai Medical College, Fudan University, Shanghai, China

**Keywords:** breast-conserving surgery, mastectomy, triple-negative breast cancer, breast cancer-specific survival, overall survival

## Abstract

Breast-conserving surgery (BCS) including radiotherapy (RT) has been demonstrated to provide at least equivalent prognosis to mastectomy in early-stage breast cancer. However, studies on triple-negative breast cancer (TNBC) patients are relatively scarce. The current population-based study aimed to investigate the distinct outcomes between BCS+RT and mastectomy in patients with TNBC. Utilizing the Surveillance, Epidemiology, and End Results (SEER) database, we enrolled 11,514 female TNBC cases diagnosed during the years 2010–2013. Those patients were subdivided into BCS+RT (5,469) and mastectomy groups (6,045), and we conducted a survival comparison between the two groups. The endpoints were breast cancer-specific survival (BCSS) and overall survival (OS). In the overall cohort, patients with BCS+RT exhibited distinctly better breast cancer-specific survival (BCSS) (log-rank, *p* < 0.001) and overall survival (OS) (log-rank, *p* < 0.001) than did mastectomy patients. When stratifying the TNBC patients according to age, histology grade, TNM stage, tumor size, and lymph node (LN) status, most patients in the BCS+RT group presented with better survival than did the patients in the mastectomy group, except for the grade I (log-rank, *p* = 0.830, both BCSS and OS) and stage I (log-rank, BCSS, *p* = 0.127; OS, *p* = 0.093) patients. In addition, after adjusting for confounding variables by multivariable Cox proportional hazard analysis, BCS+RT still tended to present with higher BCSS and OS. In conclusion, from our study on SEER data, BCS+RT displayed elevated BCSS and OS in TNBC patients compared to mastectomy, at least equally. Our study provided further evidence for surgeons that BCS with RT is available for TNBC patients.

## INTRODUCTION

Breast cancer is the most common cancer among women worldwide. The mortality rate is declining in many high-income countries [1] due to the application of multidisciplinary approaches and the management of breast cancer with effective individualized treatments, such as hormone treatments, chemotherapy, radiotherapy and surgery. However, we should strive to pursue better treatments for breast cancer.

Currently, breast cancers are classified into four distinct subtypes: luminal A, luminal B, human epidermal growth factor receptor 2 (HER2)-overexpressing, and triple-negative breast cancer (TNBC) [2]. Among these types, TNBC characterized by the absence of estrogen receptor (ER) and progesterone receptor (PR) and no HER2 overexpression accounts for 10–20% of invasive breast cancers and shares a considerable overlap with BRCA1-mutated tumors [3, 4]. Among BRCA1-associated tumors, 75%–85% are also characterized as the TNBC subtype. In addition, TNBC tends to exhibit a more aggressive nature, a metastatic pattern, and poorer prognosis compared with other subtypes.

Regarding surgical treatments, breast-conserving surgery (BCS) including postoperative radiotherapy (RT) and mastectomy are available in current practice. Increasing evidence shows that BCS+RT is at least equal with mastectomy in terms of outcome [5–11]. As summarized by van Maaren et al. in a population-based study published in Lancet Oncology, both the randomized controlled trials and observational studies showed equal or improved survival for BCS+RT compared with mastectomy in highly selected patients. Additionally, a recent population-based study in the Netherlands described a 10-year survival comparison between BCS+RT and mastectomy patients with T1-2N0-1M0 breast cancer and found improved overall survival (OS) and breast-relative survival in the BCS+RT group, although the 10-year distant metastasis-free survival rates were equivalent in other subgroups, except for the T1N0 subgroup [10]. However, for TNBC patients, the outcomes between BCS and mastectomy remain controversial. Prior to Abdulkarim's estimation of 768 TNBC patients registered in a single institution in 2011, there had been a need for a more aggressive surgical approach for TNBC patients, as there is higher locoregional recurrence (LRR) in TNBC after BCS. Abdulkarim et al. found that women with T1-2N0 TNBC treated with BCS had a lower risk of LRR than did those treated with modified radical mastectomy without RT [5]. A subsequent study consisting of a total of 1,325 patients with TNBC showed improved five-year LRR-free survival rates, distant metastasis-free survival and OS in the BCS group [11]. These studies indicated that TNBC might not be considered a contraindication for breast conservation. However, these studies were limited by their relatively small sample sizes and their single-center populations.

Considering the poor prognosis and aggressive characteristics of TNBC patients and the controversy on whether BCS+RT instead of mastectomy is the surrogate for improved survival, we aimed to conduct comparisons of BCSS and OS between mastectomy and BCS+RT for TNBC patients utilizing Surveillance, Epidemiology, and End Results (SEER), a large population-based database.

## RESULTS

### Demographics and clinical characteristics of the study population

In this study cohort, 13,753 female patients diagnosed with primary TNBC between 2010 and 2013 from the SEER data-based registry were eligible. Of these, we excluded 509 who did not receive surgical therapy and 1,730 who did not receive radiotherapy after BCS. Therefore, the final study population of the cohort consisted of 11,514 patients with non-metastatic TNBC. All of these patients were stratified by surgical treatment (Table 1), with patients undergoing BCS+RT (5,469 of 11,514 patients; 47.5%) and mastectomy (6,045 of 11,514; 52.5%). The median follow-up time was 22 months. Table 1 outlines the major baseline characteristics of the research cohort. Except for marital status, notable differences were detected in all relevant clinical and pathological variables between the two surgical type groups. Compared with the patients who underwent mastectomy, the BCS group presented a high percentage of older women (75.0% VS. 60.6%; *P* < 0.001), and their lesions tended to present a more benign biological phenotype, such as better differentiation (grade I and II, 20.5% VS. 17.5%; *P* < 0.001), small tumor size (T1, 59.1% VS. 35.9%; *P* < 0.001), lower probability of lymph node (LN) metastasis (N0-1, 95.7% VS. 86.4%; *P* < 0.001), and earlier TNM stage (stage I, 52.8% VS. 28.1%; *P* < 0.001). In addition, the BCS group had a higher proportion of patients of black race (20.2% VS. 18.7%, *P* = 0.002) and was much more likely to have a higher proportion of left laterality (52.7% VS. 50.6% *P* = 0.024).

**Table 1:** Clinicopathological features of TNBC patients in the study population

Characteristics		BCS+RT (*n* = 5469)		Mastectomy (*n* = 6045)		Total (*n* = 11,514)		*P^c^*
		No	%	No	%	No	%	
**Median follow-up (months) (IQR)**		23 (12–35)		20 (10–33)		22 (11–34)		
**Median age (years) (IQR)**		58 (50–65)		53 (44–63)		56 (47–64)		
**Age (years)**	**20–49**	1365	25.0	2380	39.4	3745	32.5	**< 0.001**
	**50–79**	4104	75.0	3665	60.6	7769	67.5	
**Race**	**White**	3987	72.9	4400	72.8	8387	72.8	**0.002**
	**Black**	1103	20.2	1129	18.7	2232	19.4	
	**Other^a^**	379	6.9	516	8.5	895	7.8	
**Marital status**	**Married**	3409	62.3	3677	60.8	7086	61.5	**0.097**
	**Not married^b^**	2060	37.7	2368	39.2	4428	38.5	
**Laterality**	**Left**	2882	52.7	3058	50.6	5940	51.6	**0.024**
	**Right**	2587	47.3	2987	49.4	5574	48.4	
**Grade**	**I**	141	2.6	104	1.7	245	2.1	**< 0.001**
	**II**	977	17.9	952	15.8	1929	16.8	
	**III and IV**	4351	79.6	4989	82.5	9340	81.1	
**AJCC stage**	**I**	2887	52.8	1697	28.1	4584	39.8	**< 0.001**
	**II**	2287	41.8	3211	53.1	5498	47.8	
	**III**	295	5.4	1137	18.8	1432	12.4	
**Tumor size (cm)**	**≤2**	3234	59.1	2168	35.9	5402	46.9	**< 0.001**
	**> 2 and ≤ 5**	2071	37.9	3038	50.3	5109	44.4	
	**> 5**	164	3.0	839	13.9	1003	8.7	
**Nodal status**	**0**	4287	78.4	3580	59.2	7867	68.3	**< 0.001**
	**1 to 3**	948	17.3	1644	27.2	2592	22.5	
	**4 to 10**	164	3.0	496	8.2	660	5.7	
	**> 10**	70	1.3	325	5.4	395	3.4	
**Radiation**	**Yes**	5469	100	1838	30.4			
	**No**	0	0	4207	69.6			

Abbreviations: BCS, breast-conserving surgery; RT, radiation therapy; IQR, interquartile range; AJCC, American Joint Committee on Cancer.

^a^Other includes American Indian/Alaskan native and Asian/Pacific Islander.

^b^Not married includes divorced, separated, single (never married), unmarried or domestic partner and widowed.

^c^*P* values for the Chi-square test were calculated between the BCS+RT and mastectomy groups; bold type indicates significance.

### Prognostic factors associated with BCSS and OS

We investigated the prognostic factors associated with BCSS and OS in the cohort of patients with TNBC. A Cox proportional hazards regression model was used for univariate and multivariate analyses of BCSS and OS in the TNBC population. The univariate Cox regression analysis of each variable is shown in Supplementary Table S1 and revealed that race, marital status, grade, AJCC stage, tumor size, LN status, and surgical types were significantly associated with BCSS and OS. After adjusting for those variables presented above in the multivariate Cox regression analysis, race, grade and AJCC stage were no longer independent prognostic factors for TNBC patients (Table 2). However, compared with grade III, grade I was an independent predictor of OS in patients with TNBC. Additionally, the clinical pathological characteristics of marital status, tumor size, and nodal status were independent factors for TNBC patients; furthermore, “not married” status, higher tumor burden, and more LN involvement were associated with poor BCSS and OS. Compared with mastectomy, RT after BCS resulted in excellent survival in patients with TNBC (hazard ratio [HR], 0.606; 95% confidence interval [95%CI], 0.502 to 0.731; *P* < 0.001 and HR, 0.579; 95%CI, 0.488 to 0.687; *P* < 0.001, for BCSS and OS, respectively).

**Table 2:** Multivariate Cox proportional hazard regression model of breast cancer-specific survival (BCSS) and overall survival (OS)

Variables		BCSS		OS	
		HRs (95% CI)	*P*^c^	HRs (95% CI)	*P*^c^
**Race**	**White**	Reference		Reference	
	**Black**	1.022 (0.842–1.241)	0.827	1.061 (0.890–1.264)	0.511
	**Other ^a^**	0.805 (0.582–1.113)	0.189	0.824 (0.613–1.108)	0.200
**Marital status**	**Married**	Reference		Reference	
	**Not married ^b^**	1.223 (1.223–1.040)	**0.015**	1.308 (1.129–1.516)	**< 0.001**
**Grade**	**I**	0.330 (0.106–1.030)	0.056	0.263 (0.085–0.820)	**0.021**
	**II**	0.865 (0.679–1.102)	0.241	0.879 (0.707–1.093)	0.246
	**III and IV**	Reference		Reference	
**AJCC stage**	**I**	Reference		Reference	
	**II**	0.695 (0.467–1.035)	0.073	0.785 (0.550–1.121)	0.183
	**III**	0.779 (0.527–1.152)	0.211	0.810 (0.561–1.169)	0.261
**Tumor size (cm)**	**≤ 2**	Reference		Reference	
	**> 2 and ≤ 5**	1.574 (1.167–2.121)	**0.003**	1.534 (1.163–2.022)	**0.002**
	**> 5**	3.165 (2.232–4.488)	**< 0.001**	2.862 (2.069–3.958)	**< 0.001**
**Nodal status**	**0**	Reference		Reference	
	**1 to 3**	2.104 (1.670–2.651)	**< 0.001**	1.902 (1.540–2.349)	**< 0.001**
	**4 to 10**	4.450 (2.832–6.993)	**< 0.001**	3.858 (2.527–5.889)	**< 0.001**
	**> 10**	8.489 (5.490–13.127)	**< 0.001**	7.528 (5.012–11.308)	**< 0.001**
**Type of surgery**	**BCS+RT**	0.606 (0.502–0.731)	**< 0.001**	0.579 (0.488–0.687)	**< 0.001**
	**Mastectomy**	Reference		Reference	

Abbreviations: AJCC, American Joint Committee on Cancer; RT, radiation therapy; BCS, breast-conserving surgery; HRs, hazard ratios; CI, confidence interval; BCSS, breast cancer-specific survival; OS, overall survival.

^a^Other includes American Indian/Alaskan native and Asian/Pacific Islander.

^b^Not married includes divorced, separated, single (never married), unmarried or domestic partner and widowed.

^c^Bold type indicates significance.

### Comparison of survival between mastectomy and BCS+RT

**Figure 1:**
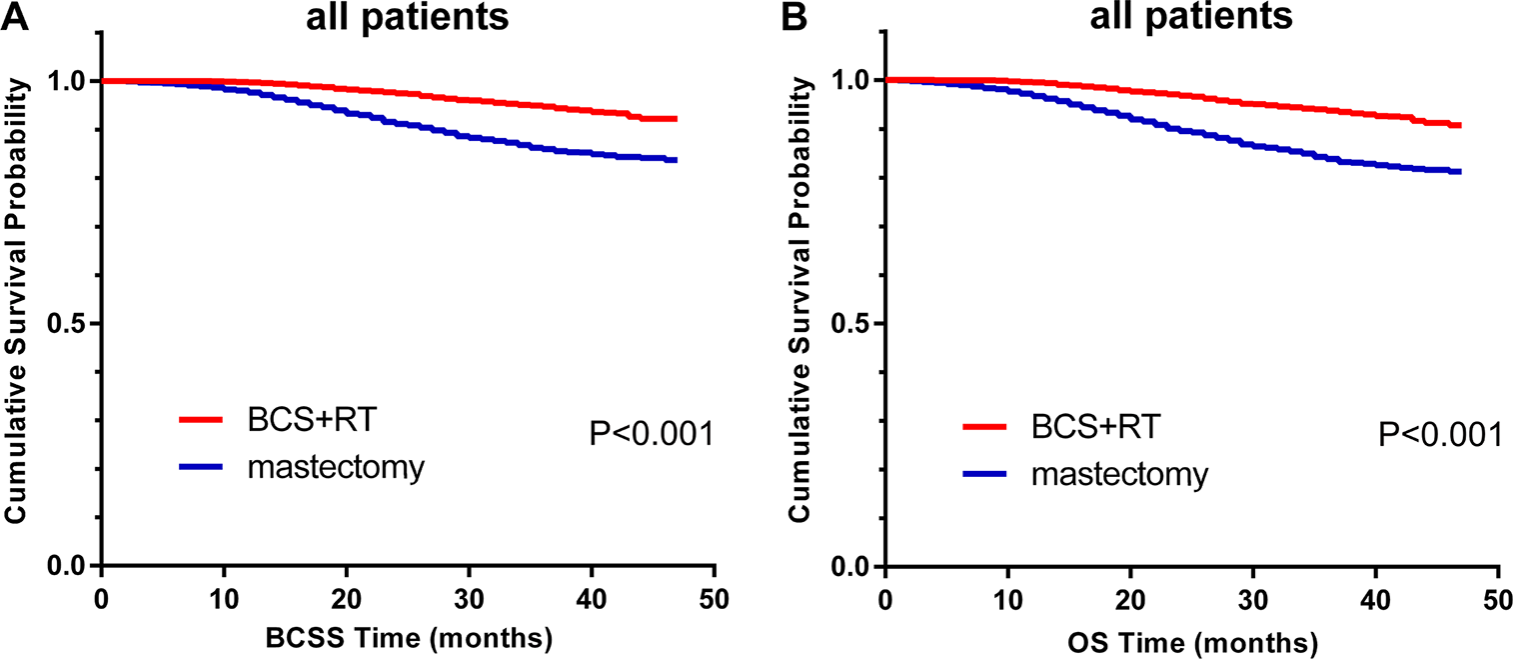
Kaplan–Meier curves of BCSS. (**A**) and OS. (**B**) by locoregional treatment for all patients; BCS+RT vs. mastectomy.

We investigated BCSS and OS in patients with TNBC treated with mastectomy compared with those receiving BCS+RT. In this study, Kaplan-Meier analysis was used to generate BCSS and OS for these two surgical types (Figure 1). The analysis indicated that patients with BCS+RT had better survival than patients with mastectomy in terms of BCSS (*P* < 0.001) and OS (*P* < 0.001). In the multivariate analysis, excellent survival was identified in the BCS+RT group when compared with the mastectomy group (HR, 0.606; 95%CI, 0.502 to 0.731; *P* < 0.001 and HR, 0.579; 95%CI, 0.488 to 0.687; *P* < 0.001, for BCSS and OS, respectively). To further investigate the probable factors influencing the survival of the two surgical types, we stratified all patients according to age, histology grade, AJCC stage, tumor sizes and LN status. As seen in Supplementary Figure S2, grade I patients showed similar survival between the two subgroups (*P* = 0.830 for both BCSS and OS). However, patients with cancer grades II, III and IV fared worse following mastectomy than patients with BCS+RT. Similarly, stage II and III patients showed improved survival with BCS+RT, while stage I patients presented the same survival rates for BCS+RT and mastectomy (Supplementary Figure S3). Additionally, better survival rates with BCS+RT were present when patients were stratified by age, tumor size and LN status, as shown in Supplementary Figures S1, S4, and S5. After multivariate adjustment, the differences between locoregional treatments in grade I (HR, 0.529; 95% CI, 0.032 to 8.626; *P* = 0.655, for both BCSS and OS) and stage I (HR, 0.743; 95% CI, 0.464 to 1.190; *P* = 0.217 and HR, 0.737; 95% CI, 0.497 to 1.095; *P* = 0.131, for BCSS and OS, respectively) were not significant (Table 3). To our surprise, univariate and multivariate analyses indicated that no subgroups of BCS+RT patients had poorer prognoses.

**Table 3:** Multivariate Cox proportional hazard regression model of breast cancer-specific survival (BCSS) and overall-survival (OS) comparing breast-conserving surgery (BCS) with mastectomy, stratified according to clinical variables

Variables^b^	BCS+RT VS mastectomy^a^			
	BCSS		OS	
	HRs (95% CI)	*P^c^*	HRs (95% CI)	*P^c^*
**Age at Diagnosis (y)**				
20–49	0.704 (0.508–0.974)	**0.034**	0.756 (0.556–1.026)	0.073
50–79	0.544 (0.432–0.684)	**< 0.001**	0.492 (0.400–0.606)	**< 0.001**
**Histologic Grade**				
I	0.529 (0.032–8.626)	0.655	0.529 (0.032–8.626)	0.655
II	0.571 (0.334–0.976)	**0.041**	0.558 (0.346–0.898)	**0.016**
III and IV	0.587 (0.480–0.718)	**< 0.001**	0.553 (0.460–0.666)	**< 0.001**
**AJCC Stage**				
I	0.743 (0.464–1.190)	0.217	0.737 (0.497–1.095)	0.131
II	0.617 (0.479–0.795)	**< 0.001**	0.560 (0.443–0.708)	**< 0.001**
III	0.513 (0.355–0.741)	**< 0.001**	0.483 (0.340–0.686)	**< 0.001**
**Tumor Size (cm)**	v			
0–2	0.715 (0.498–1.027)	0.069	0.690 (0.504–0.946)	**0.021**
2–5	0.572 (0.447–0.731)	**< 0.001**	0.528 (0.420–0.664)	**< 0.001**
> 5	0.566 (0.335–0.954)	**0.033**	0.525 (0.317–0.869)	**0.012**
**LN Status**				
negative	0.696 (0.523–0.926)	**0.013**	0.626 (0.487–0.804)	**< 0.001**
1–3 positive	0.553 (0.382–0.745)	**< 0.001**	0.532 (0.389–0.727)	**< 0.001**
4–9 positive	0.590 (0.352–0.991)	**0.046**	0.536 (0.325–0.885)	**0.015**
> 9 positive	0.482 (0.260–0.892)	**0.020**	0.445 (0.247–0.801)	**0.007**

Abbreviations: BCSS, breast cancer-specific survival; OS, overall survival; HRs, hazard ratios; CI, confidence interval; LN Status, lymph node status.

^a^Using mastectomy as a reference.

^b^Adjusted using a multivariate Cox proportional hazard regression model, including age, race, marital status, grade, AJCC stage, tumor size, LN status.

^c^ Bold type indicates significance.

## DISCUSSION

To our knowledge, this study is the first using SEER data to examine the different outcomes between BCS+RT and mastectomy for TNBC patients. In our study, we found that BCS+RT could improve BCSS and OS compared with mastectomy. Furthermore, upon stratifying the TNBC patients according to age, histology grade, stage, tumor size, and LN status, most patients with BCS+RT presented with better survival than did patients with mastectomy, except for the grade I and stage I patients, who had the same survival in the BCS+RT and mastectomy groups. These findings indicated that BCS+RT is at least equivalent to mastectomy in terms of BCSS and OS.

The finding that the long-term survival of early-stage breast cancer patients treated with BCS+RT is at least equivalent to treatment with mastectomy has been demonstrated in several prospective and retrospective randomized controlled trials [7–10, 12, 13]. Recently, a Dutch population-based study conducted a comparison of 10-year OS and breast relative survival between BCS+RT and mastectomy for patients with early breast cancer (T1–2, N0–1, M0), which further confirmed the availability of BCS+RT [10]. However, these studies did not analyze the different outcomes between BCS+RT and mastectomy for TNBC patients. Furthermore, it was not observed that T1-2N0 TNBC treated with mastectomy without RT exhibited a significant increased risk of LRR compared with those treated with BCS until 2011 in a study from a cancer registry at a single institution [5]. Additionally, most studies on locoregional treatment of TNBC patients have been limited by relatively small sample sizes and have demonstrated inconsistent outcomes. Adkins et al. identified a total of 1325 patients with TNBC who underwent BCS or mastectomy and found that the five-year LRR-free survival and distant metastasis-free survival rates were higher in the BCS group [11]. A cohort study including 1,138 Asian TNBC patients who were treated with BCS, mastectomy alone or mastectomy plus RT showed that for 775 T1-2N0-1M0 TNBC patients, the adjusted risks of mortality in the three groups were not significantly different [14]. However, our study consisted of 11,514 TNBC patients, constituting a larger cohort and a wide range of patients from the SEER database, and provided more convincing evidence that BCS+RT may not be contraindicated for TNBC patients. Additionally, our primary outcomes of BCSS and OS can represent the ultimate effects of different surgical types.

Our result that patients with BCS+RT exhibited improved OS and BCSS in TNBC may be associated with the baseline characteristics between two groups and the application of RT in the BCS group. Considering baseline characteristics, we stratified the whole patient population according to age, grade, and T, and N stages, and most patients in the BCS+RT group presented with better survival than did patients in the mastectomy group, except for the grade I and stage I patients. Furthermore, we observed that 69.6% of patients underwent mastectomy without RT in our study. Thus, we suspected that the BCS+RT was favored over mastectomy may due to RT. And accumulating evidence shows that radiation can induce an abscopal effect by stimulating the immune system to inhibit distant metastasis lesions [15–20]. Additionally, we recognized that the BRCA1 mutation in TNBC patients might influence our results. A relevant study indicated that tumors lacking functional BRCA1 were highly radiosensitive [21]. Therefore, for TNBC patients, who share a considerable overlap in BRCA1 mutation, in the context of BCS, RT to the breast and surrounding tissue could eradicate recessive BRCA1-deficient tumor lesions and thereby decrease LRR [22]. However, to date, with no consistent evidence available, the significance of RT for BCS requires further exploration with large-scale prospective studies.

In our study, there were 164 cases of 1003 tumors larger than 5 cm in size among TNBC patients accepting BCS+RT; those patients showed superior survival compared to those in the mastectomy group. This finding seemed discordant with the National Comprehensive Cancer Network (NCCN) guidelines that tumors larger than 5 cm in size are at high risk of recurrence for patients with BCS+RT. However, over the past several decades, neoadjuvant therapy (NAT) has proven beneficial for locally advanced breast cancer, as it renders inoperable tumors operable or downstages them, thus increasing the rates of BCS. In a large national database of 5,685 patients with T3 primary tumors, 15.6% of whom received BCS, similar survival rates were found for BCS and mastectomy [23]. Furthermore, Bhoo-Pathy et al. [14] suggested that BCS with RT was significantly associated with a lower mortality risk than was mastectomy without RT for 363 T3-4, N2-3, M0 TNBC patients. Therefore, we speculated that BCS+RT could also be available after NAT in advanced TNBC patients, although information on NAT was absent from our study.

One of the strengths of our study rests on the sizable number of triple-negative breast cancer patients in the SEER database, which ensures the strength and objectivity of our conclusions. Inevitably, our study had several limitations. In terms of follow-up data, it is a well-known fact that information regarding Her-2 expression in the SEER database was not available until 2010. Therefore, we were compelled to focus on the short-term survival outcomes after initial diagnosis and to identify any outcome-related factors; in this context, an inadequate follow-up duration may lead to skewed results. However, concerning TNBC subtype, the early peaks of recurrence and mortality occur within the first 2–3 years after diagnosis. Additionally, information on adjuvant or neoadjuvant chemotherapy not available for our study and probably unknown variables of tumor biology that we are still not aware of may exert a certain influence on our results.

In conclusion, from our study on SEER data, BCS+RT displayed elevated BCSS and OS in TNBC patients compared to mastectomy, at least equally. Although cosmetic impairments resulting from mastectomy can be addressed with immediate reconstruction, we still should consider the benefits of improved outcomes and an avoidable deterioration in quality life during the surgical decision-making process. Therefore, BCS+RT is a preferable choice for TNBC patients if given adequate adjuvant treatment.

## MATERIALS AND METHODS

### Ethics statement

For access to the SEER database, informed consent was not required, but a Data-Use Agreement for the SEER 1973–2013 Research Data File was completed.

### Patients

The SEER 18 registry research database was utilized to generate a case listing with a total of 13,753 eligible patients according to the following criteria: female, age at diagnosis (20–79), year of diagnosis (2010–2013), race (white, black, other), marital status at diagnosis, site recode (breast), unilateral, histological grades (I to IV), AJCC stages (I-III), T1-T3, N0-N3, M0, no lymph-vascular invasion, breast subtype (TNBC), the first and only malignant primary tumor, surgical treatment, record of radiation therapy, cause of death, and survival (months). We excluded patients with no explicit type of surgery listed and patients who received BCS without RT. In addition, SEER cause-specific deaths classified as not first tumors and patients with comorbidities were excluded. Additionally, we did not include patients with lymph-vascular invasion and patients with *in situ* disease and metastatic breast cancer at the time of presentation. Finally, 11,514 cases were enrolled in our study.

Of particular note, surgeries with primary site codes of 20–24 were categorized as receiving BCS; surgeries with codes of 30–80 were categorized as receiving mastectomy. The primary outcomes of our study were BCSS and OS. BCSS was defined as the time from the date of diagnosis to the date of death due to breast cancer or the last follow-up, and OS was measured from the date of diagnosis to the date of death due to all causes (including breast cancer) or the last follow-up.

### Statistical analysis

Statistical analysis was performed using the SPSS version 20.0 software package (IBM SPSS Statistics, Chicago, IL, US). The following variables were analyzed: age, race, marital status, laterality, grade, AJCC stage, tumor size, LN status, and treatment (BCS+RT vs. mastectomy). The differences in clinical characteristics between the two groups (BCS+RT and mastectomy) were examined using the Chi square test. The BCSS and OS survival curves were estimated using the Kaplan-Meier method, and survival differences were assessed using the log-rank test. The Cox proportional hazards regression model was conducted on univariate and multivariate analyses of BCSS and OS in the TNBC population. Univariate Cox regression analysis was performed for each prognostic variable, and those variables with *P* < 0.05 were included in the multivariate Cox model analysis. All reported *P* values are two-sided, and differences were considered statistically significant when *P* < 0.05.

## SUPPLEMENTARY MATERIALS TABLE AND FIGURES


