# Supplementary material for: The different outcomes between breast-conserving surgery and mastectomy in triple-negative breast cancer: a population-based study from the SEER 18 database
Source: Oncotarget. 2016 Dec 16;8(3):4773–80. doi: 10.18632/oncotarget.13976 (PMC5354870; doi:10.18632/oncotarget.13976)
Supplement: Supplementary file 1 [file oncotarget-08-4773-s001.pdf]

The different outcomes between breast-conserving surgery and mastectomy in triple-negative breast cancer: a population-based study from the SEER 18 database

Supplementary Materials

Supplementary Table S1: Univariate Cox proportional hazard model of breast cancer-specific survival (BCSS) and overall survival (OS)

Variables		BCSS		OS	
		HRs (95%CI)	P	HRs (95%CI)	*P*
**Age (years)**	**20-49**	1.098 (0.932–1.293)	0.263	0.956 (0.821–1.114)	0.568
	**50–79**	Reference		Reference	
**Race**	**White**	Reference		Reference	
	**Black**	1.231 (1.021–1.485)	**0.030**	1.286 (1.085–1.524)	**0.004**
	**Other a**	0.860 (0.622–1.188)	0.360	0.872 (0.649–1.172)	0.363
**Marital status**	**Married**	Reference		Reference	
	**Not married b**	1.357 (1.161–1.587)	**< 0.001**	1.452 (1.259–1.675)	**< 0.001**
**Laterality**	**Left**	Reference		Reference	
	**Right**	0.926 (0.792–1.082)	0.334	0.967 (0.839–1.115)	0.646
**Grade**	**I**	0.191 (0.061–0.593)	**0.004**	0.160 (0.052–0.498)	**0.002**
	**II**	0.666 (0.524–0.846)	**0.001**	0.692 (0.557–0.859)	**0.001**
	**III and IV**	Reference		Reference	
**AJCC stage**	**I**	0.286 (0.222–0.370)	**< 0.001**	0.342 (0.275–0.426)	**< 0.001**
	**II**	Reference		Reference	
	**III**	3.785 (3.206–4.467)	**< 0.001**	3.586 (3.074–4.182)	**< 0.001**
**Tumor size (cm)**	**≤ 2**	Reference		Reference	
	**> 2 and ≤ 5**	2.933 (2.390–3.599)	**< 0.001**	2.637 (2.201–3.160)	**< 0.001**
	**> 5**	8.356 (6.635–10.523)	**< 0.001**	7.076 (5.746–8.713)	**< 0.001**
**Nodal status**	**0**	Reference		Reference	
	**1 to 3**	3.005 (2.481–3.639)	**< 0.001**	2.633 (2.213–3.134)	**< 0.001**
	**4 to 10**	6.052 (4.790–7.648)	**< 0.001**	5.215 (4.203–6.470)	**< 0.001**
	**> 10**	13.121 (10.431–16.504)	**< 0.001**	11.481 (9.311–14.156)	**< 0.001**
**Treatment**	**BCS+RT**	0.347 (0.291–0.415)	**< 0.001**	0.351 (0.298–0.413)	**< 0.001**
	**Mastectomy**	Reference		Reference	

Abbreviations: AJCC, American Joint Committee on Cancer; RT, radiation therapy; BCS, breast-conserving surgery; HRs, hazard ratios; CI, confidence interval; BCSS, breast cancer-specific survival; OS, overall survival.
[a]Other includes American Indian/Alaskan native and Asian/Pacific Islander.
[b]Not married includes divorced, separated, single (never married), unmarried or domestic partner and widowed.

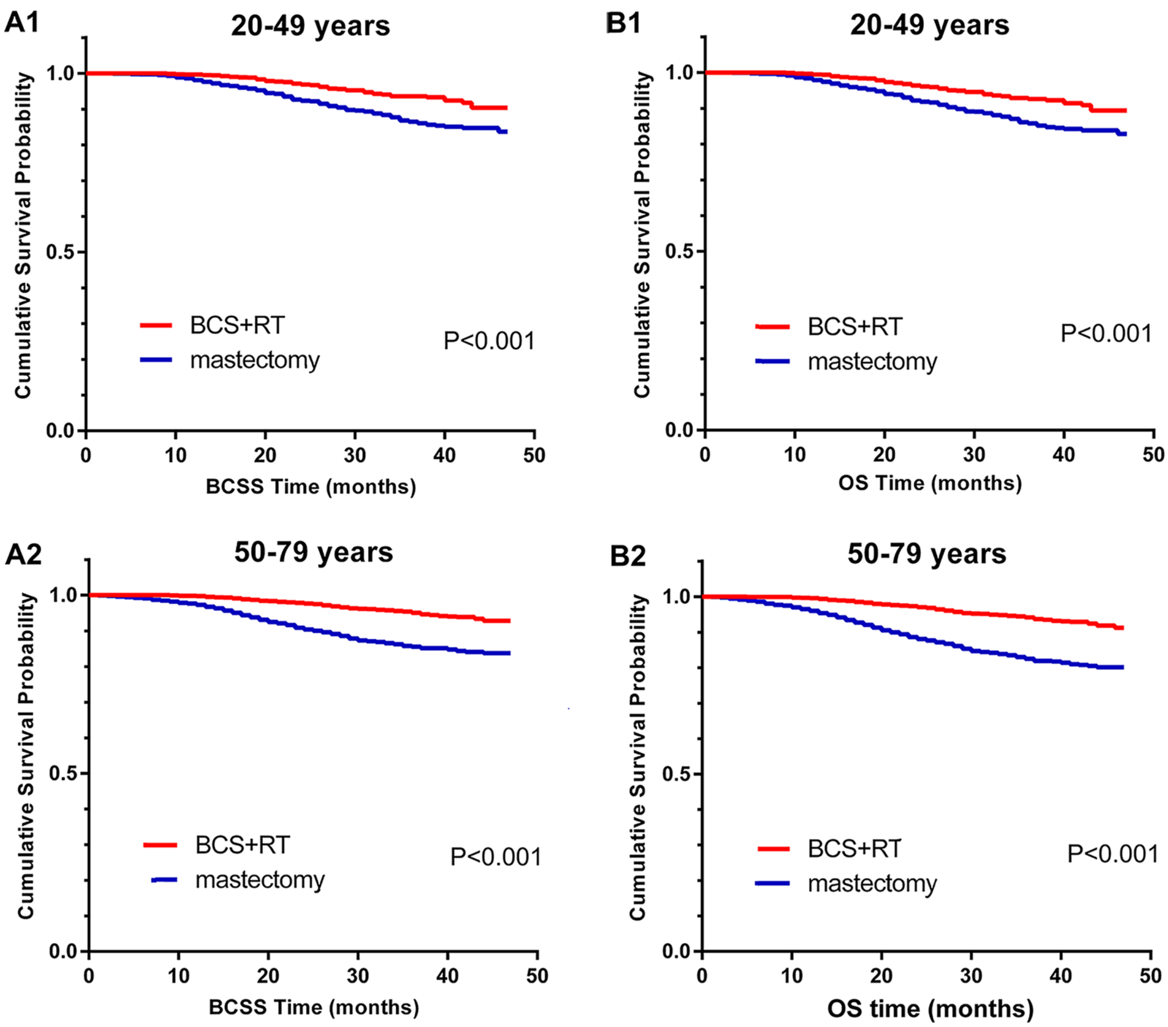

Supplementary Figure S1: Kaplan–Meier curves of BCSS. (**A**) and OS. (**B**) by locoregional treatment for all patients stratified by age: 20–49 (1) and 50–79 (2); BCS+RT vs. mastectomy.

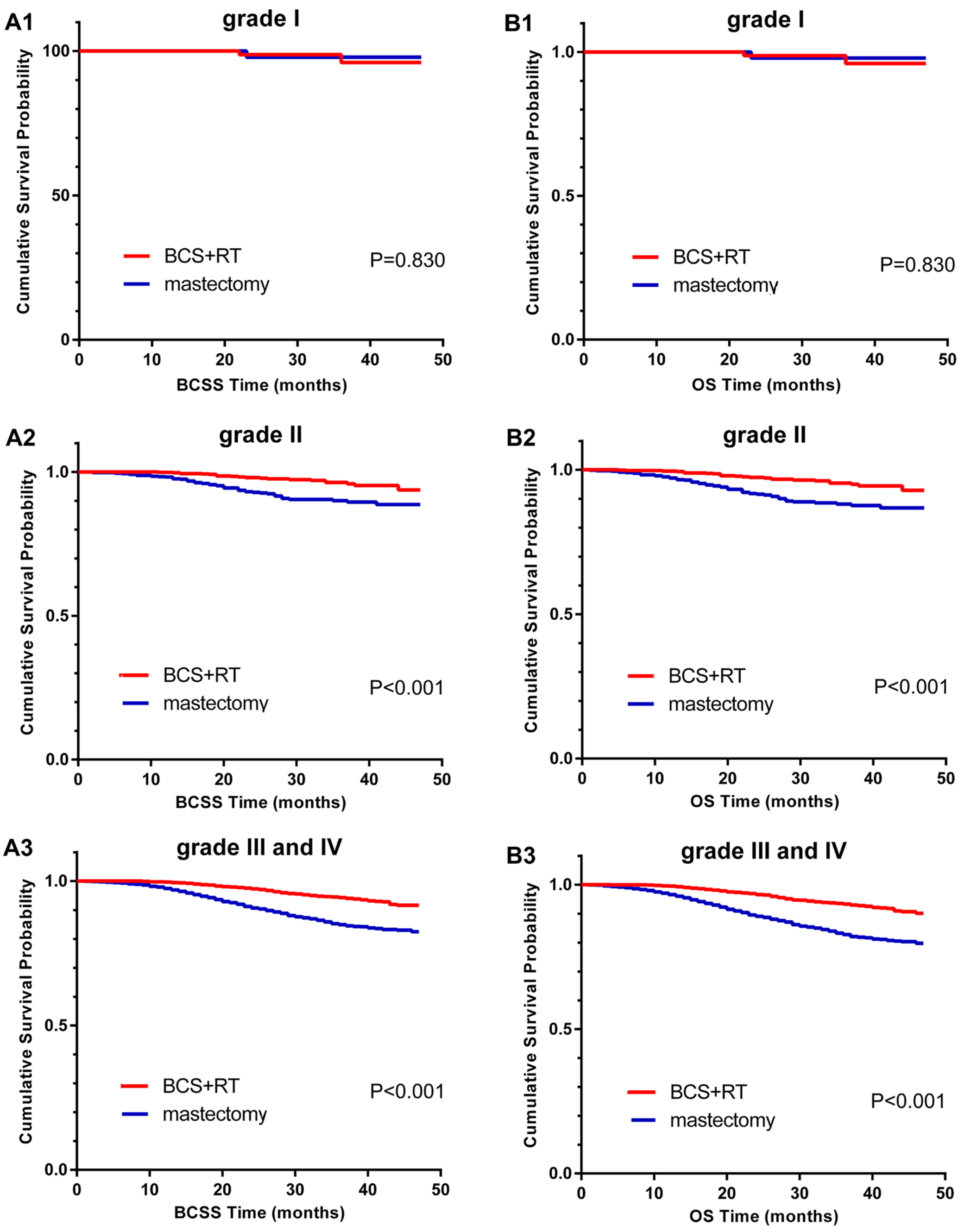

Supplementary Figure S2: Kaplan–Meier curves of BCSS. (**A**) and OS. (**B**) by locoregional treatment for all patients stratified by grade I (1), II (2) and III and IV (3); BCS+RT vs. mastectomy.

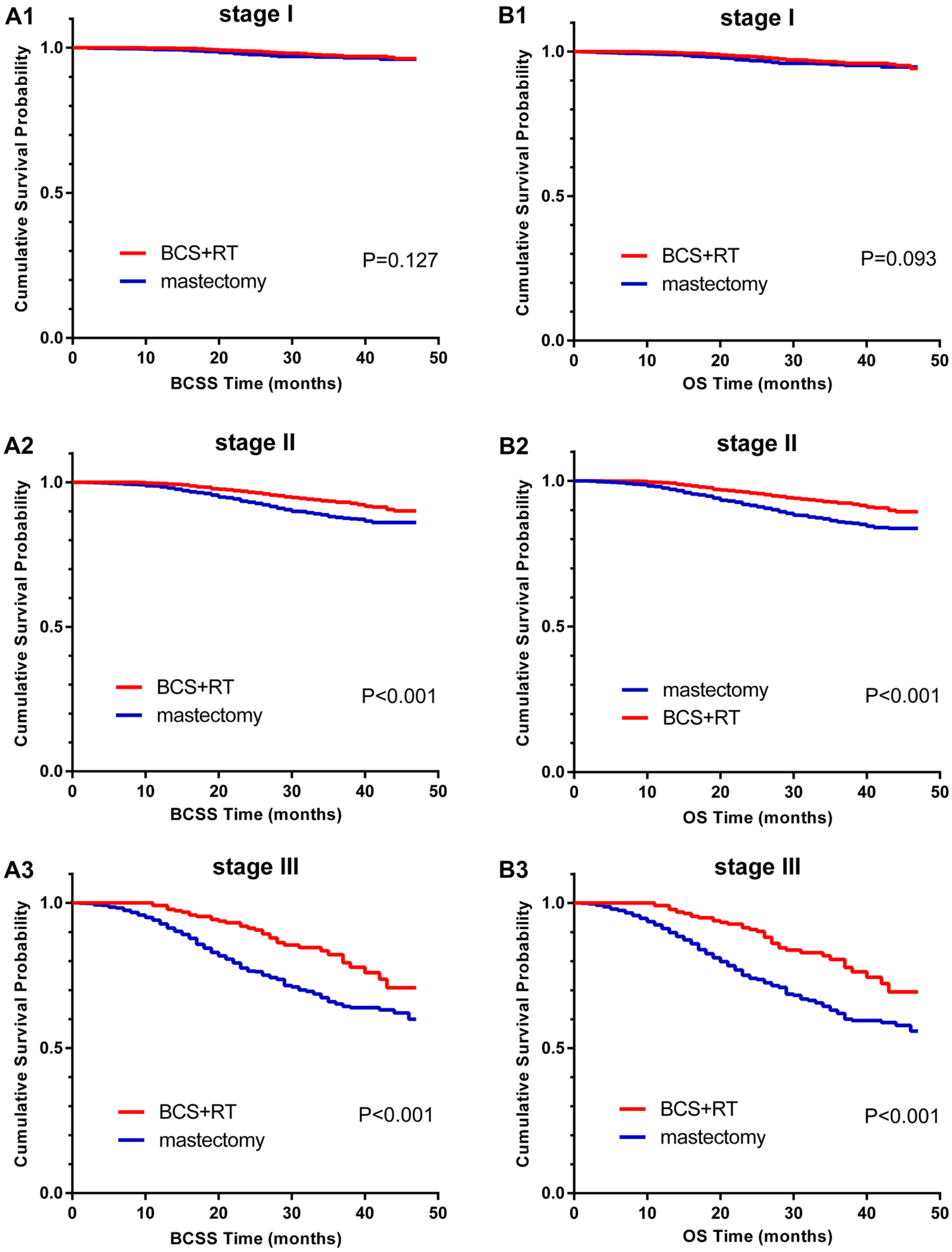

Supplementary Figure S3: Kaplan–Meier curves of BCSS. (**A**) and OS. (**B**) by locoregional treatment for all patients stratified by stage I (1), II (2) and III (3); BCS+RT vs. mastectomy.

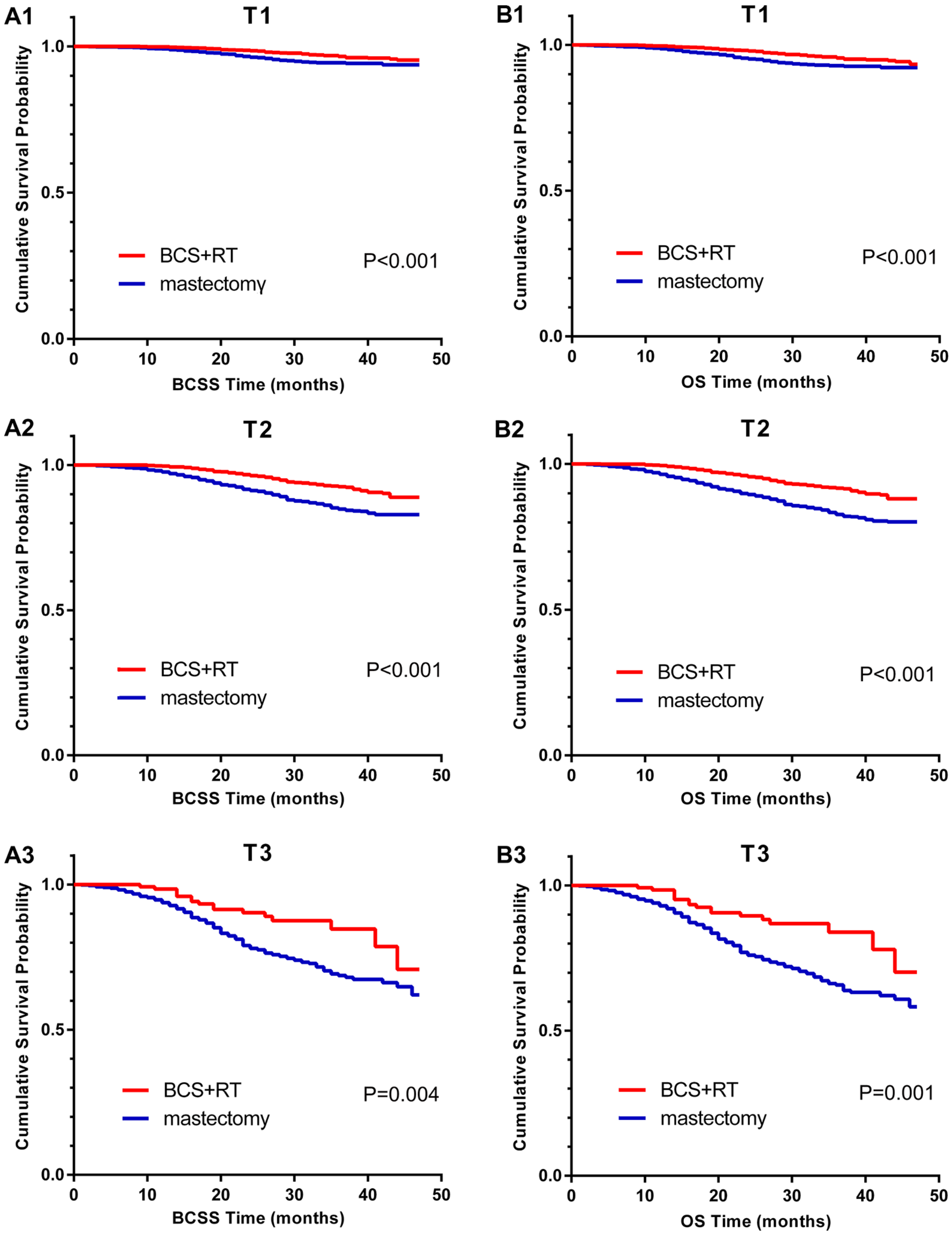

Supplementary Figure S4: Kaplan–Meier curves of BCSS. (**A**) and OS. (**B**) by locoregional treatment for all patients stratified by T1 (1), T2 (2) and T3 (3); BCS+RT vs. mastectomy.

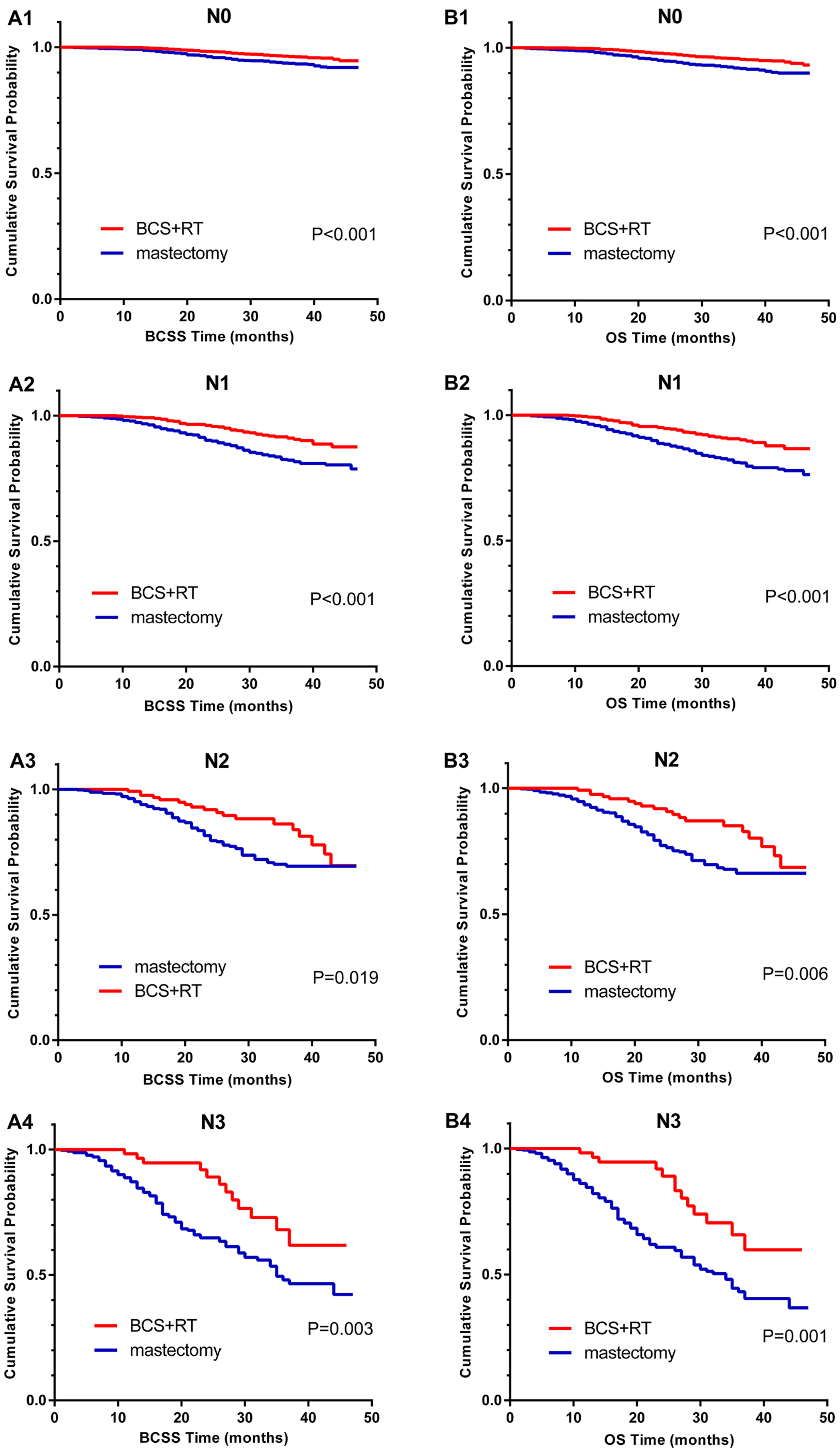

Supplementary Figure S5: Kaplan–Meier curves of BCSS. (**A**) and OS. (**B**) by locoregional treatment for all patients stratified by N0 (1), N1 (2), N2 (3) and N3 (4); BCS+RT vs. mastectomy.
